# Personal values clusters and their associations to social media behaviors and psychological well-being

**DOI:** 10.1186/s40359-024-02046-4

**Published:** 2024-10-08

**Authors:** Claudiu Gabriel Ionescu, Ella Magdalena Ciuperca, Adriana Cotel, Monica Licu

**Affiliations:** 1 Carol Davila, University of Medicine and Pharmacy, Bucharest, Romania; 2 National Institute for Research & Development in Informatics, University of Bucharest, Bucharest, Romania

**Keywords:** Personal values, Social media, Algorithms, Well-being

## Abstract

The current study contributes to the literature by assessing the associations between personal values, explored with Schwartz`s Portrait Values Questionnaire, social media behaviors, assessed with Bergen Scale of Social Media Addiction and Social Media Motivations to Use Scale and psychological well-being assessed with Patient Health Questionnaire-4, in a sample of first-year medical students. It was examined medical students’ personal values profiles and the perceived influence of social media on self-aspects. All participants (*N* = 151) were Romanian and English module students, young (average age = 19.2, SD = 1.5), 68,9% females and 31,1% males. Pearson coefficient correlation analyses were performed to verify the associations between the main four clusters of personal values (Self Transcendence, Self Enhancement, Openness to change and Conservationism) with social media behaviors and psychological wellbeing. The most frequent cluster of values was Self-Transcendence (M = 5.21) while the least was Self-Enhancement (M = 4.05). There was no significant correlation between social media addiction, psychological wellbeing and a specific cluster of values while the perception of self-aspects influenced by social media included involvement in community problems, creativity for Openness to change group (*R* = .24;.22, *p* < .05), tolerance towards sexual minorities and self-evaluation in Self Transcendence group (*R* = .24;.21;.42, *p* < .05) while Conservationism and Self Enhancement groups didn`t report any change. The findings highlight the need for awareness and education of medical students and general population in the field of Digital Ethics including social media complex impact on personal values as AI-algorithms may imply a potential destabilization and perpetual shaping of one`s behavior with still unpredictable individual and societal effects.

**Supplementary Information:**

The online version contains supplementary material available at 10.1186/s40359-024-02046-4.

## Introduction

### Social media behavior and psychological well-being

Social media have become a meeting place of decision for most aspects of users` lives, from choosing a profession, a life partner, a life philosophy, a political ideology, and a spiritual trend to areas such as entertainment and paying administrative taxes, and there are no signs that things might stop moving at this fast pace. This led to the reflection on the overwhelming impact that these platforms can have on decisions and, not least, on personal identity and values.

Social media are defined as “web or mobile technologies that create interactive platforms through individuals or communities that share, create, discuss and modify user-generated content” [1]. An immersive environment in which users’ lives are fully or partially exposed represents more than just the need for basic human interaction. Currently, there are more than 4.7 billion users on the planet, with 10.1% growth per year, which means there are 1 million new users per day and 59% of the planet population [2]. Locally, 67.3% of the Romanian population uses social media daily, with 46.8% of Romania using TikTok, a platform with 12% of its users growing in the last year. In terms of platform types, TikTok is the most growing, with more than 1 billion users [3]. Second, Instagram is an app used daily by more than 2 billion users who upload visual content, and the average age is 30 years. Additionally, a mean of 100,000 years in duration would last to watch all the content uploaded on the app in only one day, which is approximately 34 million videos [4].

Moreover, social media addiction has been correlated with numerous psychopathological variables, such as depression, anxiety, suicidal ideation and feelings of loneliness, dating fatigue, information fatigue, relationship fatigue due to an immensity of offers, and a reduction in meaningful social connections, especially among young people [5–7], as well as with beneficial effects such as connection, community membership, personal expression and social support [8]. Approximately 4,69% of total social media users globally are estimated to be addicted with ratings increasing up to 13% for severe levels and 25% for moderate levels with university students reporting rates of 18,4% [9, 10].

Addiction is maintained through engagement by algorithms, which can be considered ambivalent, malleable and generative but also autonomous. Additionally, recommender systems are seen as traps requiring engagement with the minds of the users and can only be effective if their creators understand and work with the target’s world view and motivations, so the autonomous agency of the users can be effectively exploited [11, 12]. Algorithmic profiling can disrupt this individual experience of personal identity, as the model of each user is continuously reconfigured based on the feedback provided by other users’ interactions with the system. This was previously conceptualized as algorithmized identity, and the networked self has been studied for several years [13–15]. Additionally, algorithms are considered a part of a cognitive process in which the brain performs some operations, while the algorithm also plays a causal role in governing an individual’s personal values and behavior as an interactive system or cognitive integration [16, 17]. Thus, algorithms through social media addiction can shape what users see, potentially influencing their beliefs and values.

### Personal values—stability and change

Values are constructs or concepts that refer not only to the "need to be good or to be well" or “to have something good in them intrinsically” but also to “what truly matters in life” [18, 19]. Additionally, values converge toward objectives that stimulate motivation and are classified in the order of importance for everyone being considered stable throughout a lifespan [20, 21].

From a psychological point of view, values are cognitive structures that affectively invest and give meaning and continuity [22] and that create an important system of priorities for the individual [23]. Schwartz’s Theory of Basic Human Values, the most recognized evidence-based system in psychology [24], brings motivational concepts underlying the system, such as values oriented toward “openness to change” and “conservationism”, where the first contains openness for change and independence and the second contains the maintenance of the same order already known and self-transcendence and self-enhancement, which are oriented toward self-fulfillment and transcendence or universal ones. According to Schartz, these theories are based on the basic human biological needs of survival and coexistence.

Personal values can change depending on the level of fulfillment of the ideal self perceived by the individual [25]. For psychological coherence and mental well-being, users may align their personal values with those of the group or vice versa according to the signals received from the environment, based on anxiety and motivation, not conflict. Thus, the change in values can only take place within a change in self-perceived identity [15].

Changing values through social media behaviors is a subject already of interest in the literature, but from more theoretical than empirical points of view [15, 26]. Users related thinking about the self, speculating that their feed says something about the type of person they are or even allows them to better understand their own social, sexual, and cultural identity [27–29]. However, until now, no studies have revealed if one’s identity and personal values are impacted by one’s social media behavior. The literature states that users` need for certainty but also lack of perspective in life may lead to polarized social cohesion, augmented by cognitive biases [30]. There is thus a fluidization of values, both individual and, especially, collective, with hyperconsciousness tendencies such as ecological or closed-circuit movements, including political, spiritual, health attitudes or cultural hyperpolarization [31–34] reinforced by social media behavior.

### Why social media behavior and personal values?

The idea that personal values are well-rooted, stable and defined concepts, and take into account the neuroplasticity of the brain seems to lose its validity with users immersed within social media algorithms, which may impact it at both the individual and group levels. This context raises complex ethical questions and debates about the long-term societal effects but also individual effects on privacy, consent, personal identity and fairness, which are at the advent of their exploration among social sciences and mental health professionals with a paucity of studies examining how these algorithms interact with and influence personal values [11, 12, 35–37].

The literature highlights that social media shapes how people see themselves, their behavior changes, and their relationships with marginalized identities [14, 26, 38]. Studies thus far have drawn vague connections between personal values and social media behaviors and have not yet established a relationship between certain value clusters and specific social media behaviors such as feed content, posting or perceived self-aspects influenced by social media [14, 38].

Despite extensive research on the effects of social media behaviors on mental health, there is a notable gap in understanding its impact on personal values. This article seeks to bridge this gap by investigating each cluster of personal values according to Schwartz’s Theory of Basic Human Values and explore its relationships with social media behaviors and psychological well-being using specific psychometric tools with a focus on self-aspects perceived as influenced by social media.

It is hypothesized that social media behaviors not only reflect users' current values but also have the potential to shape and modify their self-aspects perceived as influenced, especially for specific clusters such as Openness to Change and Self-Transcendence. Also, it is expected that each cluster of values would be positively associated with specific behaviors corresponding to their description, with higher social media addiction scores potentially being associated with values such as hedonism, achievement, and power and low values of benevolence and universalism, without a specific link to psychological well-being; moreover, the predominant cluster of values would be self-transcendence, considering previous studies on medical students, and there would be associations between social media addiction scores and psychological well-being but no specific link to a certain cluster of values.

## Methods

A cross-sectional study using an online survey was performed to explore the personal values dimensions, psychological well-being, social media addiction level and its usage patterns including motives, perceptions regarding influence and feed structure in a sample of 188 Romanian and English module medical undergraduate students. The online survey was hosted on SurveyMonkey and was promoted from March to May 2023 on courses, google classroom, where no form in incentives were offered to potential participants. The online survey was expected to be completed in approximately 25 min, on average. All participants were informed about the nature of the study and required to provide informed consent online before taking the study while they could complete the survey at their convenience. They were also provided with all the information required about the ethical aspects of the study. The nationality of the English module students was not explored in the survey due to ethical, potential for bias and privacy considerations.Moreover, to be eligible for partaking in the study all the participants were required to be aged at least 18 years old. The present research has been granted with the ethical approval of the “Carol Davila” University of Medicine and Pharmacy Ethics Committee no. 14873/2023. A total of 37 participants were excluded from the present study due to mis checking the attention item resulting a sample of 151 participants (19.2 SD = 1.5) of which 68,9% were women.

## Measures

Sociodemographic data were evaluated with questions regarding age, gender, type of module attended, year of study, place of origin and a highlight on social circle, activities with the group of friends, family or colleagues.

### Personal values

Moral values dimensions were assessed using the 21 item Portrait Value Questionnaire, English version, previously published (PVQ-21) [39] which assesses the importance given to the four higher-order value domains (Conservationism, Openness to change, Self Enhancement and Self Transcendence) by the participants. Conservationism values emphasize self-restraint, established customs, and stability, whereas openness to change values emphasize change, independence, and freedom. The second dimension shows the struggle between Self Enhancement values, which emphasize one's own interests and relative success and domination, and Self Transcendence values, which emphasize others' welfare and interests. Schwartz further classified these value dimensions by focus (personal vs. societal) and direction (self-protective vs. self-expansive). Personal values include Self Enhancement and Openness to change, whereas social values include self-transcendence and conservation. The instrument consists of 21 verbal portraits of a person and his/her objectives or aspirations, which reflect the importance of a value. Example of items are: “Thinking up new ideas and being creative is important to him/her. He/She likes to do things in his/her own original way” and “He/She strongly believes that people should care for nature. Looking after the environment is important to him/her” which assesses personal openness to change and self-transcendence values. Respondents self-reported their similarity to each portrait using a 6-point Likert scale (1 = not like me at all; 6 = very much like me). The higher the similarity score to the person described, the higher the importance assigned to that specific value. The PVQ-21 exhibits high internal consistency, with Cronbach's α values ranging from 0.80 to 0.92 across different studies and populations [40]. All the internal consistency coefficients were above the minimum acceptable threshold of 0.7 [40], except Cronbach’s Alpha of the PVQ-21 subscales. As mentioned, we consider its results unreliable given the skew data of the mentioned subscales, and, therefore, we take as reference the McDonald’s Omega and the Greatest Lower Bound coefficient, which are remarkably close to the 0.7 threshold even for PVQ-21 subscales.

### Social media behaviors

Next, we continued the questionnaire with items regarding the average time spent on various social media platforms the participants having the possibility to choose from 5-point Likert scale (1 = 0-1 h; 2 = 1,01-2 h; 3 = 2,01-3 h; 4 = 3,01-4 h and 5 = 4,01-5 h). We asked participants to check their personal feed information checking on how often they find a specific content on their personal feed choosing from 6-point Likert scale as follows: 1 – very rarely; 2 – rarely; 3 – neither rarely nor often; 4 – often; 5 – very often; 6 – don`t know/don`t answer. Furthermore, we continued with questions about the favorite content participants post using the same 6-point Likert scale previously described and a question focusing on how often they post on social media having to choose from 5 variables (several times a day, once a day, once every 2–3 days, at approximately one week, less than once a week).

Furthermore, there were explored the motivations for using social media using SMU-SNS (The Scale of Motives for Using Social Networking Sites) which is an instrument used to measure the different reasons people use social networking sites (SNS). It was developed and validated by Petergral (2019) [41]. Internal consistency (Cronbach's α) of the SMU-SNS factors ranges from 0.77 to 0.90, indicating excellent reliability. The construct validity of the SMU-SNS has been confirmed via factor analysis, which revealed multiple dimensions representing distinct motives for using SNS. Also, content validity was ensured during the development process, while additionally, the criterion validity of the scale was established by correlating its scores with other established measures of similar constructs [41–43].

These dimensions along with their definitions were included: motives related to finding a romantic partner, dating or having sexual encounters, making new friends and meeting new people, motives related to obtaining information and help on academic matters such as exams, class notes, or group assignments, motives related to feeling part of society through online social networks., motives related to following and looking for details of the lives of ones´ friends through their profiles and publications, entertainment, social recognition, self-expression, information, Each of the 27 items is rated on a Likert scale from 1 to 7, with higher scores indicating stronger agreement with the reason being rated. The SMU-SNS has been used in a variety of research studies to investigate the relationships between SNS use and various psychological and social factors [41–43].

### Self-aspects perceived as changed

It were also explored the perceptions of participants regarding the degree social media changed several self-aspects such as independence, tolerance towards sexual minority groups, creativity, the way of socialization, perception of security and more (see Supplementary Materials) using the same 7-point Likert scale previously described.

### Social media addiction

Regarding social media addiction, it was used The Bergen Social Media Addiction Scale [44]; English version for English module students and Romanian version, previously validated [45]. This is a 6-item self-report scale used to assess the levels of problematic social media use, in line with the components model of behavioral addiction [46]. The BSMAS is measuring social media addiction according to the core components of addiction (i.e., salience, mood modification, tolerance, withdraw symptoms, conflict, and relapse), without a specific timeframe. Items are rated on a five-point Likert scale, from 1 (“very rarely”) to 5 (“very often”). Higher scores indicate higher levels of problematic social media use. The total score of the English version was used in this research [47] and showed acceptable internal consistency in the present sample (α = 0.74). Internal consistency (Cronbach's α) of the BSMAS is high, ranging from 0.82 to 0.92 across studies [45]. In this study the internal consistency coefficients were above the minimum acceptable threshold of 0.7 [40].

### Psychological well-being

PHQ-4 (Patient Health Questionnaire-4) scale is an international brief screening scale for anxiety and depressive symptoms [48] and has been used widely in the general population [49–51] and various clinical samples [52, 53]. This scale assessed four symptoms of mental distress of the respondents over the past 2 weeks. An example item was “feeling down, depressed or hopeless.” The items were answered on a 4-point Likert scale from 0 = “not at all” to 3 = “nearly every day.” The total PHQ-4 score has a theoretical range from 0 to 12, with higher scores denoting greater distress. The internal consistency (Cronbach's α) of the PHQ-4 is high, ranging from 0.80 to 0.92 across studies [53].

### Statistical analysis and data analytic strategy

It was first described the demographics of our participants. Then, it was assessed the internal consistency of all our scales, namely PHQ-4, SMU-SNS, PVQ-21 and the Bergen scale. As such, we calculated Cronbach’s Alpha. However, given that four items of PVQ-21 were severely skew (< -1 or > 1), and seven others were moderately skew (< -0.5 or > 0.5), we decided to calculate the McDonald’s Omega and Greatest Lower Bound coefficients too. These two coefficients are recommended for skew data because, in contrast with the canonical Cronbach’s Alpha, they offer unbiased estimates of internal consistency [54–58]. For correlation analyses involving ordinal scales it was employed the Spearman coefficient, while for numerical scales we used Pearson coefficient. All reported correlations are bivariate. Data management and descriptive analyses were performed using JASP statistical software, version 0.17.2 statistical software, and SPSS version 23 [55].

## Results

The sociodemographic characteristics are presented in Table 1 while it was found that the majority of participants reported having 2 friends (29.1%), followed by 3 friends (23.8%). Only 6.0% of participants reported having no close friends, and 13.2% reported having 5 or more close friends. When considering good friends, however, the majority of participants reported having 5 or more (64.9%). The number of participants reporting no good friends was very small (1.3%). When it comes to family most of the participants reported engaging in common activities with their family members 'sometimes' (35%). However, a substantial portion of the sample rarely engage in common activities with their family (25.2%). In the context of colleagues, the highest proportion of participants reported engaging in activities 'often' (38.4%). Still, a sizable number of participants indicated they 'sometimes' (29.1%) or 'rarely' (21.2%) engage in common activities with their colleagues. As for friends, more than half of the participants (52.3%) reported often participating in common activities with their friends. This was followed by 'sometimes' (27.8%), indicating a relatively high level of engagement with friends. Across all three categories, the number of participants who never participate in common activities is low (1.3% for family, 3.3% for colleagues, and 0.7% for friends), suggesting that most participants have some level of social interaction within these groups as seen in Supplementary Materials.

**Table 1:** Sociodemographic characteristics of the study participants

Characteristic	n	%
**Year**		
First	104	68.9
Second	47	31.1
**Place of origin**		
Not specified	1	0.7
Rural	21	13.9
Urban	129	85.4
**Module**		
Not specified	2	1.3
Romanian module	137	90.7
English module	12	7.9
**Gender**		
Female	104	68.9
Male	43	28.5
Prefer not to say	2	1.3
Other	2	1.3

*n* = *151*

Data regarding average time spent on social media is presented in in Table 2.

**Table 2:** What is the average time you spend on the following social networks?

	**TikTok**	**Instagram**	**WhatsApp**	**YouTube**	**LinkedIn**	**Twitter**	**Facebook**	**Others**
0–1 h	85 (56.3%)*	52 (34.4%)	44 (29.1%)	56 (37.1%)	146 (96.7%)	143 (94.7%)	131 (86.8%)	112 (74.2%)
1,01–2 h	29 (19.2%)	56 (37.1%)	59 (39.1%)	40 (26.5%)	1 (0.7%)	4 (2.6%)	15 (9.9%)	25 (16.6%)
2,01–3 h	22 (14.6%)	35 (23.2%)	26 (17.2%)	26 (17.2%)	-	-	3 (2.0%)	8 (5.3%)
3,01–4 h	9 (6.0%)	7 (4.6%)	11 (7.3%)	12 (7.9%)	-	-	-	1 (0.7%)
4,01–5 h	5 (3.3%)	1 (0.7%)	11 (7.3%)	17 (11.3%)	-	1 (0.7%)	-	2 (1.3%)
Total	151 (100.0%)	151 (100.0%)	151 (100.0%)	151 (100.0%)	151 (100.0%)	151 (100.0%)	151 (100.0%)	151 (100.0%)

^*^Numbers in parentheses indicate percentages

Considering the description of personal feed pages on social media we found that entertainment (46%), close friends post (37,7%), educational information (29,8%) and fashion trends (21.9%) was “very often” checked while “very rarely” were checked items like dating (57%), sexual content (49%), live streaming (47,6%) and religious information (43%). Additional information may be found in the Supplementary Material data.

Regarding posting on social media and its frequency we found that the highest frequency of "several times a day" is observed on WhatsApp, with over half of the participants (50.3%) reporting this level of usage. LinkedIn and Twitter show similar patterns, with the majority of participants (96.0%) posting less than once a week. The pattern for Facebook and YouTube is like LinkedIn and Twitter, but with slightly less frequency in the "less than once a week" category. Instagram displays a more evenly distributed posting frequency, although "less than once a week" is still the dominant category. Lastly, TikTok and other platforms show diverse usage patterns, but "less than once a week" remains the most common frequency. This data reveals that WhatsApp is the most frequently used platform for daily posting among the studied participants, whereas LinkedIn and Twitter are used least frequently. Regarding the favorite type of content posted we found among the “often” and “very often” checked items entertainment (19,4%), memes (24,8%) and volunteering and social causes (15,9%) while in the category of “very rarely” and “rarely” we found live streaming (87,4%), sexual (89,4%) and political comments (85,5%). More data is available in Supplementary Materials.

Another data regarding the perceived self-aspects social media changed in the last two years revealed that ability to concentrate (29,8%), tolerance towards sexual minority groups (30,5%) and creativity (25,8%) were highly scored as changed while no or very minor change was checked among tolerance of religious minority groups (47,1%), involvement in online communities (47,1%) and selection of mentors (45,7%). More data is available in Supplementary Materials.

In the clusters of personal values, Self Transcendence scored the highest average (M = 5.21), while Self Enhancement scored the lowest (M = 4.05) as seen in Table 3. All the internal consistency coefficients were above the minimum acceptable threshold of 0.7 [40], except Cronbach’s Alpha of the PVQ-21 subscales. As mentioned, we consider its results unreliable given the skew data of the mentioned subscales, and, therefore, we take as reference the McDonald’s Omega and the Greatest Lower Bound coefficient, which are remarkably close to the 0.7 threshold even for PVQ-21 subscales (data in Supplementary Materials).

**Table 3:** Descriptive Statistics for the study variables

Variables	M	SD	Range	Cronbach’s Alpha	McDonald’s Omega	Greatest Lower Bound
Bergen Total	15.24	5.62	6.00–30.00	.85	.85	.88
PHQ Total	6.09	3.73	0.00–12.00	.88	.88	.93
**Motives for Social Media Usage**						
Romantic Purpose	1.81	1.15	1.00–5.33	.85	.86	.86
New Friendships	2.75	1.53	1.00–7.00	.94	.94	.94
Academic Purposes	4.45	1.76	1.00—7.00	.92	.92	.92
Social Connectedness	4.26	1.80	1.00—7.00	.9	.9	.9
Following Others	3.82	1.60	1.00—7.00	.81	*NaN*	.86
Entertainment	4.98	1.59	1.00—7.00	.87	.89	.89
Social Recognition	2.04	1.14	1.00—6.00	.71	.72	.72
Self-Expression	2.86	1.56	1.00—7.00	.84	.84	.84
Seeking Information	4.86	1.56	1.00—7.00	.86	.87	.87
**Clusters of Personal Values**						
Self-Transcendence	5.21	0.57	3.08—6.00	.57	.57	.69
Self-Enhancement	4.05	0.87	1.75—6.00	.65	.66	.69
Openness to Change	4.62	0.66	2.83—6.00	.64	.67	.77
Conservatorism	4.12	0.76	2.00—5.83	.62	.63	.7

*M* Mean, *SD* Standard Deviation, *Range* Minimum—Maximum

The relationship between personal values as per Schwartz's theory and various social media-related variables was examined using Pearson's correlation coefficient (see Table 4 for full details). Significant relationships were noted between the Self-Transcendence and Romantic Purpose, the Openness to Change cluster of values and New Friendships, the Self Enhancement with Romantic purpose, following others, social recognition and entertainment while Self-Enhancement/Conservationism clusters and several motives for using social media. The strength and direction of these correlations varied. There were no significant correlations between any personal value cluster and psychological wellbeing or social media addiction scores as seen in Table 4.

**Table 4:** Correlations between personal values and social media variables

Variable	Self-Transcendence	Self-Enhancement	Openness to Change	Conservationism
Social Media Addiction	-.123	.113	.030	-.035
Anxiety and Depression	-.008	-.009	-.073	.078
**Motives for using social media**				
Romantic Purpose	-.183*	.310**	.184*	-.289**
New Friendships	-.014	.084	.278**	-.295**
Academic Purposes	.109	.029	.075	-.156
Social Connectedness	.052	.183*	.074	-.249**
Following Others	.098	.229**	.065	-.299**
Entertainment	-.013	.175*	.037	-.171*
Social Recognition	-.072	.223**	.203*	-.305**
Self-Expression	.137	.109	.071	-.241**
Seeking Information	.072	-.036	.056	-.073

All reported correlations are Pearson coefficients

^*^p < .05, **p < .01

Table 5 presents the Spearman correlation coefficients between personal values and perceived changes of self over the past two years. Notably, Conservationism showed significant negative correlations with ability to collaborate, involvement in online communities, involvement in community problems, evaluation of self, independence, tolerance of religious minorities, tolerance towards sexual minorities, and way to bond friends. For the other personal values, there were a few significant correlations found. The ability to be creative and involved in community issues was positively correlated with Openness to change. Self-transcendence showed a significant positive correlation with self-evaluation and tolerance towards sexual minorities.

**Table 5:** Correlations between personal values and perceived changes of self in the last two years

Items	Self-Transcendence	Self-Enhancement	Openness to Change	Conservationism
Ability to collaborate	.145	.063	.152	-.235**
Ability to concentrate for a long time	.065	.133	.034	-.149
Creativity	.052	-.077	.240**	-.160
Involvement in online communities	.141	.084	.064	-.237**
Involvement in community problems	.144	-.005	.239**	-.314**
Evaluation of self	.248**	.099	.000	-.209**
Independence	.066	.081	.088	-.163*
Perception of security	.032	.070	.088	-.141
Selection of mentors	.063	.037	.083	-.156
Time spent in offline relationships	.142	.021	-.048	-.048
Tolerance of religious minorities	.088	.014	.100	-.169*
Tolerance towards sexual minorities	.211**	-.008	.108	-.252**
Way of eating	.009	.015	.159	-.147
Way of making decisions	.017	.085	.032	-.084
Way of socialization	.057	.061	.037	-.081
Way to bond friends	.077	.055	.182*	-.229**

All reported correlations are Spearman coefficients

^*^p < .05, **p < .01

### Correlates of psychological well-being and motives for social media usage

As illustrated in Table 6, there were high correlations between anxiety and depression scores and social media addiction. Additionally, anxiety and depression scores were positively associated with academic purposes and entertainment motives for usage SM.

**Table 6:** Correlations between anxiety and depression (PHQ-4) and personal values, addiction to social media, motives for using SNS and perceived changes in the last two years (Spearman and Pearson)

Variables	Anxiety and Depression^a^	Common Activities with^b^		
		Colleagues	Family	Friends
**Cluster of Values**				
Self-Transcendence	-0.008	-.239**	-0.152	-0.075
Self-Enhancement	-0.009	0.034	-0.017	-0.007
Openness to Change	-0.073	.206*	-0.134	0.132
Conservationism	0.078	-0.056	.236**	-0.035
**Social Media Addiction**	.437**	-0.062	-0.112	-0.075
**Anxiety and Depression**	—	-.166*	-0.064	-0.135
**Motives for using SNS**				
Romantic Purpose	-0.077	.207*	-.166*	0.127
New Friendships	-0.029	.227**	-0.097	0.076
Academic Purposes	.211**	.196*	0.032	-0.008
Social Connectedness	0.084	.165*	0.003	0.029
Following Others	0.062	0.045	-0.067	0.156
Entertainment	.218**	0	-0.157	-0.003
Social Recognition	0.087	0.104	-0.12	0.014
Self-Expression	0.105	0.022	0.024	0.052
Seeking Information	0.06	0.049	-0.032	-0.047

^a^All reported correlations are Pearson coefficients

^b^All reported correlations are Spearman coefficients

^*^p < .05. **p < .01

## Discussions

To the best of our knowledge, this is the first study to test the relationship between basic human values designed by Schwartz’s theoretical model [59] and social media behaviors and psychological wellbeing among medical undergraduate students.

The hypothesis that social media behaviors not only reflect users' current values but also have the potential to shape and modify their self-aspects perceived as influenced was confirmed, especially for Self-Transcendence, Openness to change with no changes in Self-Enhancement and negative correlations in Convervationism. Also, the expectation that each cluster of values would be positively associated with specific behaviors corresponding to their description was true but higher social media addiction scores or modified psychological well-being were not associated with either value cluster while there are studies that highlight that a better orientation toward values is associated with superior wellbeing. The same study showed that the idea of unhealthy or conflicting values is scientifically not yet validated, as it creates theoretical discrepancies [46]. Moreover, the predominant cluster of values would be self-transcendence, as we hypothesized.

In terms of demographics, the sample typical participant included was a female, first-year medical student, enrolled in the Romanian Module, living in an urban setting. Assesing the social environment of participants considering Displacement Theory, as the more time spent on social media, the less time the liability to spend socializing with peers and family [56]. Overall, the distribution of good friends is less even than that of close friends, suggesting a pattern where individuals have a small number of close friends and a larger network of good friends. A sizable number of participants indicated that they 'sometimes’ or 'rarely' engage in common activities with their colleagues. Nevertheless, for friends, more than half of the participants reported often participating in common activities with their friends, which has already been reported to be associated with fewer psychological symptoms and overall wellbeing among the general population and medical students [60, 61].

Entertainment and seeking information as main motives to use social media consistent with previous research, and connectedness and peer support, which has been reported as well as its relationship with less or greater psychological distress depending on actively searching for new connections or maintaining existing connections [62, 63]. It was found that entertainment, close friends’ posts, educational information and fashion trends were “very often” checked which is consistent with previous literature [62].

Shorter amount of time spent on Twitter and LinkedIn than Instagram and TikTok was in accordance with previous studies [64]. This could be due to a variety of factors, such as the nature of the platforms, user demographics, or individual preferences [65]. Additionally, the proportion of users spending more than 3 h on any of these platforms is generally low, suggesting that these platforms may have features or content that encourage longer usage periods, as previously reported [66].

Worryingly but expected and in accordance with previous studies [67, 68], depression and anxiety scores showed that medical students might present moderate clinically relevant symptomatology with strong correlations between anxiety and depression scores and social media addiction as well as between academic purposes and entertainment motives for the use of social media which can be interpreted as indicating that greater academic pressure may lead to entertainment searching as a coping factor for distress-provoking factors, as previous reported [69, 70].

In previous studies, medical students’ personal values have been found to be associated with moral development [71]. In this sample, self-transcendence scored the highest, with self-enhancement scoring the lowest, which is in line with previous literature concerning medical students’ personal values framed as desire to help, altruism, patient-centered values, empathy or healthcare practitioners’ value scale such as spirituality, critical thinking or capability [72, 73]. Additionally, higher values of responsibility, truth, hedonism and hierarchy were reported among medical students [74]. In this study, the strength and direction of these correlations varied. Even if clusters such as self-enhancement are more prone to social media addiction considering self-orientation, expression or achievement through active participation in social media, there is still a need for more studies to determine how and why specific clusters of values relate to social media addiction while considering potential mediating factors such as personality traits, social support and cultural background.

Even so, self-transcendence showed a significant positive correlation with changes perceived in the last two years as affected by social media regarding self-evaluation and tolerance toward sexual minorities, which is consistent with studies previously published in the Romanian population [75] and internationally [76]. Significant negative relationships were noted between self-transcendence and romantic purpose, such as motivations to use social media, while there was a significant positive correlation with educational information and volunteering or social causes as feed received, which was expected because this construct was associated with proneness to altruism, empathy and voluntary activities [72, 77].

Self-enhancement was previously associated with low preoccupation with impression management and social desirability [78], empathy [72] and greater academic performance [79]. This study showed, interestingly, that self-enhancement dimension was negatively correlated with content related to volunteering or social causes, as the value is linked to high microworry and low macroworry [38]. Significant relationships were observed between self-enhancement and motivations to use social media for romantic purposes, social recognition and entertainment, which is in accordance with previous reported characteristics of this cluster [38] but no correlation with perceived self-aspects as changed by social media.

There is currently evidence of openness to change concerning decision-making abilities in organizations for managers, highlighting the need for value-based decision making and ethical approaches and awareness [80]. It was found that the ability to be creative and to be involved in community issues were positively correlated, and there were significant relationships between openness to change and new friendships. Considering its independence of thought, action, and feelings and readiness for change, our results are a means of optimism regarding future doctors’ values [59].

Conservationism showed significant negative correlations with various items, including ability to collaborate, involvement in online communities, involvement in community problems, evaluation of self, independence, tolerance of religious minorities, tolerance toward sexual minorities, and way to bond friends, as its main characteristics, such as security, conformity and tradition, order, self-restriction, preservation of the past, and resistance to change, are consistent with our results [59]. Finally, conservationism showed a significant negative correlation with the presence of content related to close friends and political comments in personal feeds. Significant negative relationships were highlighted with social connectedness, social recognition, entertainment or self-expression, and time spent on social media platforms.

## Strengths and limitations

This study has some limitations that we should consider. In terms of design, it was a cross-sectional study involving a single country group of Southeastern Romanian medical undergraduate students and a little proportion of English module students. Also, the study's findings are limited by the small sample size, self-selection bias as the participation was voluntary and dependent on individuals` willingness to engage and considering the specific characteristics of the sample, it may not be representative of the broader population, thereby potentially affecting the generalizability of the results. There is need for future longitudinal, using larger samples from other years of study and academic institutions and why not, general population, which should explore whether and to what extent the personal values are linked to social media behaviors and, most important, if they shift over the course of their studies along with repeated assessments. However, there were used reliable validated instruments which proved to be statistically robust thus opening potential ways for future collaborations, nationally and internationally. Methodological development of this type of studies could include direct assessment of user`s profiles activity on social media, qualitative methods, big data extraction and analysis from the main social media platforms to assess the megatrends and their impact at an individual level. Our question regarding self-aspects perceived as changed by social media was a first step into assessing, maybe, the most important aspect this study purposed. Furthermore, this is the first study to focus on social media behaviors and psychological wellbeing in undergraduate medical students’ personal values using a worldwide theoretical framework- an approach which may start promising future steps.

## Conclusions

The study suggests that, beginning their medical education, medical students scored highest on Self Transcendence while Self Enhancement scored lowest, results found to be consistent with literature emphasizing altruistic and empathic approaches as well as patient-centered and universalism values even if there is literature reporting medical schools may value self-oriented personal values encouraging students to adopt achievement and success prone behaviors. We found the expected social media behaviors according to each cluster of values characteristics and no significant association between social media addiction and psychological well-being with different clusters although we found that psychological well-being is negatively correlated with social media addiction, as expected.

Given that social media is a key factor in human connectedness, megatrends and self-perceived values and identities we found among the perceived changes influenced by social media being ability to collaborate, social causes involvement in the Openness to change cluster, self-evaluation and tolerance among sexual minorities in the Self Transcendence cluster with no change perceived in Conservationism and Self Enhancement cluster. There is need for awareness among them and their teachers regarding the potential impact social media algorithms could have on personal values as complex ethical reflections should help future doctors chose their priorities and values aligned with their professional status but, ultimately, their impact on patients.

Considering this study as a first glimpse into the complex relationship between personal values and social media the awareness could be the main aim of an era where personal values are challenged.

## Supplementary Information

Supplementary Material 1.

Supplementary Material 2.

Supplementary Material 3.

Supplementary Material 4.

Supplementary Material 5.

## Data Availability

No datasets were generated or analysed during the current study.

## Appendix 1

**Table 7:** Full correlation matrix of personal values and perceived changes of self in the last two years (Part 1)

	Conservationism	Openess to Change	Self-Enhancement	Self-Transcendence	Ability to collaborate	Ability to concentrate for a long time	Creativity	Evaluation of self	Independence	Involvement in community problems
Conservationism	—									
Openess to Change	-.66**	—								
Self-Enhancement	-.31**	-.17*	—							
Self-Transcendence	-.2*	-0.03	-.42**	—						
Ability to collaborate	-.23**	0.15	0.06	0.15	—					
Ability to concentrate for a long time	-0.15	0.03	0.13	0.07	.23**	—				
Creativity	-0.16	.24**	-0.08	0.05	.44**	.21**	—			
Evaluation of self	-.2**	0	0.1	.24**	.5**	.32**	.38**	—		
Independence	-.16*	0.09	0.08	0.07	.48**	.33**	.38**	.54**	—	
Involvement in community problems	-.31**	.23**	-0.01	0.14	.53**	.27**	.51**	.44**	.39**	—

*N* = 151. **p* < .05. ***p* < .01

**Table 8:** Full correlation matrix of personal values and perceived changes of self in the last two years (Part 2)

	Conservationism	Openess to Change	Self-Enhancement	Self-Transcendence	Ability to collaborate	Ability to concentrate for a long time	Creativity	Evaluation of self	Independence	Involvement in community problems
Involvement in online communities	-.23**	0.06	0.08	0.14	.47**	.27**	.23**	.58**	.43**	.56**
Perception of security	-0.14	0.09	0.07	0.03	.48**	.38**	.39**	.52**	.4**	.43**
Selection of mentors	-0.16	0.08	0.04	0.06	.51**	.35**	.36**	.5**	.46**	.58**
Time spent in offline relationships	-0.05	-0.05	0.02	0.14	.41**	.36**	.33**	.48**	.52**	.34**
Tolerance of religious minorities	-.16*	0.1	0.01	0.09	.46**	.26**	.28**	.5**	.43**	.58**
Tolerance towards sexual minorities	-.25**	0.11	-0.01	.21**	.49**	.27**	.3**	.49**	.54**	.55**
Way of eating	-0.15	0.16	0.02	0.01	.50**	.24**	.53**	.49**	.4**	.43**
Way of making decisions	-0.08	0.03	0.09	0.02	.54**	.3**	.35**	.59**	.74**	.5**
Way of socialization	-0.08	0.04	0.06	0.06	.58**	.37**	.43**	.54**	.6**	.46**
Way to bond friends	-.22**	.18*	0.06	0.08	.58**	.3**	.39**	.45**	.5**	.52**

*N* = 151. **p* < .05. ***p* < .01

**Table 9:** Full correlation matrix of personal values and perceived changes of self in the last two years (Part 3)

	Involvement in online communities	Perception of security	Selection of mentors	Time spent in offline relationships	Tolerance of religious minorities	Tolerance towards sexual minorities	Way of eating	Way of making decisions	Way of socialization	Way to bond friends
Involvement in online communities	—									
Perception of security	.46**	—								
Selection of mentors	.59**	.53**	—							
Time spent in offline relationships	.48**	.52**	.45**	—						
Tolerance of religious minorities	.45**	.36**	.51**	.4**	—					
Tolerance towards sexual minorities	.44**	.33**	.44**	.33**	.79**	—				
Way of eating	.37**	.51**	.46**	.37**	.23**	.21**	—			
Way of making decisions	.52**	.43**	.53**	.48**	.56**	.55**	.43**	—		
Way of socialization	.5**	.46**	.57**	.54**	.39**	.44**	.48**	.6**	—	
Way to bond friends	.51**	.39**	.47**	.39**	.44**	.45**	.39**	.58**	.69**	—

*N* = 151. **p* < .05. ***p* < .01

## Appendix 2

**Table 10:** Correlation matrix: anxiety and depression (PHQ-4), personal values, social media addiction, SNS motives, and perceived changes in the last two years (Part 1)

	Personal Values				Social Media Addiction	Anxiety and Depression	Motives for Using Social Networking Sites		
	Self-Transcendence	Self-Transcendence	Openness to Change	Conservationism			Romantic Purpose	New Friendships	Academic Purposes
Self-Transcendence	—								
Self-Transcendence	-.48**	—							
Openness to Change	-0.06	-.16*	—						
Conservationism	-.22**	-.32**	-.68**	—					
Social Media Addiction	-0.12	0.11	0.03	-0.03	—				
Anxiety and Depression	-0.01	-0.01	-0.07	0.08	.43**	—			
Romantic Purpose	-.18*	.31**	.18*	-.28**	0.1	-0.08	—		
New Friendships	-0.01	0.08	.27**	-.29**	0.11	-0.03	.47**	—	
Academic Purposes	0.11	0.03	0.08	-0.16	0.15	.21**	-0.03	.2*	—

*N* = 151. **p* < .05. ***p* < .01

**Table 11:** Correlation matrix: anxiety and depression (PHQ-4), personal values, social media addiction, SNS motives, and perceived changes in the last two years (Part 2)

	Personal Values				Social Media Addiction	Anxiety and Depression	Motives for Using Social Networking Sites		
	Self-Transcendence	Self-Transcendence	Openness to Change	Conservationism			Romantic Purpose	New Friendships	Academic Purposes
Social Connectedness	0.05	.18*	0.07	-.24**	.25**	0.08	.26**	.33**	.46**
Following Others	0.1	.22**	0.06	-.29**	.25**	0.06	.31**	.29**	.3**
Entertainment	-0.01	.17*	0.04	-.17*	.37**	.21**	0.11	0.11	.34**
Social Recognition	-0.07	.22**	.2*	-.3**	.27**	0.09	.46**	.4**	0.12
Self-Expression	0.14	0.11	0.07	-.24**	.16*	0.1	.21**	.35**	.39**
Seeking Information	0.07	-0.04	0.06	-0.07	0.15	0.06	0.07	.17*	.42**
Family	-0.15	-0.02	-0.13	.23**	-0.11	-0.06	-.16*	-0.1	0.03
Colleagues	-.23**	0.03	.2*	-0.06	-0.06	-.16*	.2*	.22**	.19*
Friends	-0.08	-0.01	0.13	-0.04	-0.08	-0.13	0.13	0.08	-0.01

*N* = 151. **p* < .05. ***p* < .01

**Table 12:** Correlation matrix: anxiety and depression (PHQ-4), personal values, social media addiction, SNS motives, and perceived changes in the last two years (Part 3)

	Motives for Using Social Networking Sites						Common Activities with:^a^		
	Social Connectedness	Following Others	Entertainment	Social Recognition	Self-Expression	Seeking Information	Family	Colleagues	Friends
Social Connectedness	—								
Following Others	.61**	—							
Entertainment	.45**	.42**	—						
Social Recognition	.29**	.35**	0.14	—					
Self-Expression	.34**	.41**	.19*	.44**	—				
Seeking Information	.49**	.33**	.43**	0.06	.22**	—			
Family	0	-0.07	-0.16	-0.12	0.02	-0.03	—		
Colleagues	.16*	0.05	0	0.1	0.02	0.05	0.12	—	
Friends	0.03	0.16	0	0.01	0.05	-0.05	.17*	.3**	—

*N* = 151. **p* < .05. ***p* < .01

^a^All reported correlations are Pearson coefficients, except for those involving the "Common Activities" variable, which are Spearman coefficients

## Abbreviations

BSMAS: Bergen Social Media Addiction Scale
PVQ-21: Portrait Value Questionnaire
PHQ-4: Patient Health Questionnaire
SM: Social Media
SMU-SNS: The Scale of Motives for Using Social Networking Sites
