# Supplementary material for: Personal values clusters and their associations to social media behaviors and psychological well-being
Source: BMC Psychol. 2024 Oct 8;12:545. doi: 10.1186/s40359-024-02046-4 (PMC11462701; doi:10.1186/s40359-024-02046-4)
Supplement: Supplementary file 3 — Supplementary Material 3. [file 40359_2024_2046_MOESM3_ESM.docx]

# Descriptive statistics

## How numerous is your group of friends?

|  | **Close Friends** | **Good Friends** |
| --- | --- | --- |
| 0 | 9 (6.0%) | 2 (1.3%) |
| 1 | 19 (12.6%) | 3 (2.0%) |
| 2 | 44 (29.1%) | 9 (6.0%) |
| 3 | 36 (23.8%) | 12 (7.9%) |
| 4 | 23 (15.2%) | 27 (17.9%) |
| 5+ | 20 (13.2%) | 98 (64.9%) |
| Total | 151 (100.0%) | 151 (100.0%) |

*Note: Numbers in parentheses indicate percentages.

Commentary:

This data provides an interesting look at the distribution of close and good friends within a sample of 151 individuals. For close friends, the majority of participants reported having 2 friends (29.1%), followed by 3 friends (23.8%). Only 6.0% of participants reported having no close friends, and 13.2% reported having 5 or more close friends.

When considering good friends, however, the majority of participants reported having 5 or more (64.9%). This suggests that while individuals may have fewer people they consider "close friends", they are more likely to have a larger number of "good friends". The number of participants reporting no good friends was very small (1.3%).

Overall, the distribution of good friends is less even than that of close friends, suggesting a pattern where individuals have a small number of close friends and a larger network of good friends.

## How often do you have activities in common with:

|  | **Family** | **Colleagues** | **Friends** |
| --- | --- | --- | --- |
| Never | 2 (1.3%) | 5 (3.3%) | 1 (0.7%) |
| Rarely | 38 (25.2%) | 32 (21.2%) | 7 (4.6%) |
| Sometimes | 54 (35.8%) | 44 (29.1%) | 42 (27.8%) |
| Often | 46 (30.5%) | 58 (38.4%) | 79 (52.3%) |
| Always | 11 (7.3%) | 12 (7.9%) | 22 (14.6%) |
| Total | 151 (100.0%) | 151 (100.0%) | 151 (100.0%) |

*Note: Numbers in parentheses indicate percentages.

Commentary:

The data presents how often a sample of 151 individuals participate in common activities with different groups - family, colleagues, and friends.

When it comes to family, the majority of the participants reported engaging in common activities with their family members 'sometimes' (35.8%). However, a substantial portion of the sample rarely engage in common activities with their family (25.2%).

In the context of colleagues, the highest proportion of participants reported engaging in activities 'often' (38.4%). Still, a sizable number of participants indicated they 'sometimes' (29.1%) or 'rarely' (21.2%) engage in common activities with their colleagues.

As for friends, more than half of the participants (52.3%) reported often participating in common activities with their friends. This was followed by 'sometimes' (27.8%), indicating a relatively high level of engagement with friends.

Across all three categories, the number of participants who never participate in common activities is low (1.3% for family, 3.3% for colleagues, and 0.7% for friends), suggesting that most participants have some level of social interaction with these groups.

## What is the average time you spend on the following social networks?

|  | **TikTok** | **Instagram** | **WhatsApp** | **YouTube** | **LinkedIn** | **Twitter** | **Facebook** | **Others** |
| --- | --- | --- | --- | --- | --- | --- | --- | --- |
| 0-1 h | 85 (56.3%) | 52 (34.4%) | 44 (29.1%) | 56 (37.1%) | 146 (96.7%) | 143 (94.7%) | 131 (86.8%) | 112 (74.2%) |
| 1,01-2 h | 29 (19.2%) | 56 (37.1%) | 59 (39.1%) | 40 (26.5%) | 1 (0.7%) | 4 (2.6%) | 15 (9.9%) | 25 (16.6%) |
| 2,01-3 h | 22 (14.6%) | 35 (23.2%) | 26 (17.2%) | 26 (17.2%) | - | - | 3 (2.0%) | 8 (5.3%) |
| 3,01-4 h | 9 (6.0%) | 7 (4.6%) | 11 (7.3%) | 12 (7.9%) | - | - | - | 1 (0.7%) |
| 4,01-5 h | 5 (3.3%) | 1 (0.7%) | 11 (7.3%) | 17 (11.3%) | - | 1 (0.7%) | - | 2 (1.3%) |
| Total | 151 (100.0%) | 151 (100.0%) | 151 (100.0%) | 151 (100.0%) | 151 (100.0%) | 151 (100.0%) | 151 (100.0%) | 151 (100.0%) |

*Note: Numbers in parentheses indicate percentages.

Commentary:

The data provides an overview of the average time spent on different social networks by a sample of 151 individuals.

For TikTok, most users spend between 0-1 hour per day (56.3%) on the platform. Instagram sees slightly more engagement with 37.1% spending between 1-2 hours and 34.4% spending 0-1 hour. WhatsApp usage is pretty evenly distributed, but slightly skewed towards 1-2 hours usage (39.1%). YouTube usage is similar to Instagram with most users spending between 0-2 hours on the platform.

LinkedIn, Twitter, and Facebook show a trend of lower usage with the majority of users spending 0-1 hour per day on these platforms (96.7%, 94.7%, and 86.8% respectively).

For "Other" social networks, the majority of users spend 0-1 hour (74.2%) per day on these platforms.

It's worth noting that the data suggests individuals tend to spend a relatively short amount of time on LinkedIn, Twitter, and Facebook compared to other platforms. This could be due to a variety of factors such as the nature of the platforms, user demographics, or individual preferences.

Also, the proportion of users spending more than 3 hours on any of

these platforms is generally low, with the exception of YouTube and WhatsApp. This suggests that these platforms may have features or content that encourage longer usage periods.

## If you would describe your PERSONAL FEED pages on social media, how often you find the following?

| **Variables** | **Very Rarely (%)** | **Rarely (%)** | **Neither Rarely nor Often (%)** | **Often (%)** | **Very Often (%)** | **Don't Know/Don't Answer (%)** |
| --- | --- | --- | --- | --- | --- | --- |
| Personal Feed: Far Away Friends | 14.6 | 16.6 | 31.1 | 21.2 | 13.2 | 3.3 |
| Personal Feed: Close Friends | 8.6 | 7.9 | 15.2 | 23.8 | 37.7 | 6.6 |
| Personal Feed: Accessing New People Profiles | 23.2 | 23.2 | 28.5 | 13.2 | 9.9 | 2.0 |
| Personal Feed: Political Comments | 31.8 | 25.2 | 12.6 | 15.9 | 11.9 | 2.6 |
| Personal Feed: Educational Information | 5.3 | 9.3 | 17.2 | 33.8 | 29.8 | 4.6 |
| Personal Feed: Health Recommendations | 8.6 | 21.9 | 23.2 | 22.5 | 17.9 | 6.0 |
| Personal Feed: Religious/Spiritual Information | 43.0 | 25.2 | 17.9 | 4.6 | 4.0 | 5.3 |
| Personal Feed: Tutorials and Guides | 10.6 | 19.9 | 20.5 | 29.8 | 15.2 | 4.0 |
| Personal Feed: Entertainment | 4.7 | 4.0 | 8.0 | 23.3 | 46.0 | 14.0 |
| Personal Feed: Fashion Trends | 19.9 | 11.9 | 19.9 | 19.2 | 21.9 | 7.3 |
| Personal Feed: Shopping | 15.9 | 18.5 | 21.9 | 16.6 | 18.5 | 8.6 |
| Personal Feed: Food Recipes | 15.3 | 15.3 | 24.0 | 24.0 | 14.7 | 6.7 |
| Personal Feed: The News of the Day | 15.3 | 17.3 | 23.3 | 23.3 | 17.3 | 3.3 |
| Personal Feed: Comments on the News of the Day | 26.0 | 22.7 | 20.0 | 16.7 | 10.7 | 4.0 |
| Personal Feed: Live Streaming | 46.3 | 22.8 | 12.8 | 10.1 | 6.0 | 2.0 |

| Influencers | 26.8 | 18.8 | 16.8 | 16.1 | 14.1 | 7.4 | 1.3 |
| --- | --- | --- | --- | --- | --- | --- | --- |
| Trends | 20.1 | 12.1 | 25.5 | 18.1 | 18.1 | 6.0 | 1.3 |
| Dating | 57.0 | 16.1 | 12.8 | 8.1 | 4.0 | 2.0 | 1.3 |
| Sexual | 49.0 | 18.8 | 14.8 | 7.4 | 4.7 | 5.4 | 1.3 |
| Volunteering/Social Causes | 20.3 | 20.3 | 27.7 | 18.9 | 9.5 | 3.4 | 2.0 |
| Tourism Recommendations | 17.6 | 21.6 | 21.6 | 20.9 | 12.2 | 6.1 | 2.0 |
| Others | 17.6 | 8.1 | 33.1 | 16.2 | 8.1 | 16.9 | 2.0 |

## What is your favorite type of content that you frequently POST on social networks?

|  |  |  |  |  |  |  |  |
| --- | --- | --- | --- | --- | --- | --- | --- |
| **Posting Topics** | **Very Rarely %** | **Rarely %** | **Neither Rarely Nor Often %** | **Often %** | **Very Often %** | **Don't Know/Don't Answer %** | **Missing %** |
| News About Yourself | 32.5 | 21.9 | 15.9 | 13.2 | 6.6 | 8.7 | 1.3 |
| Political Comments | 79.5 | 6.0 | 4.0 | 0.7 | 2.0 | 6.1 | 2.0 |
| Educational Information | 47.0 | 12.6 | 17.9 | 8.6 | 4.6 | 8.1 | 1.3 |
| Health Recommendations | 55.6 | 13.9 | 13.9 | 5.3 | 2.0 | 7.4 | 2.0 |
| Religious/Spiritual Information | 78.8 | 6.0 | 5.3 | 0.7 | 1.3 | 6.1 | 2.0 |
| Tutorials and Guides | 72.8 | 8.6 | 6.0 | 4.0 | 2.0 | 4.7 | 2.0 |
| Entertainment | 40.4 | 11.3 | 17.9 | 9.3 | 11.3 | 8.1 | 2.0 |
| Fashion Trends | 66.2 | 12.6 | 6.0 | 4.0 | 5.3 | 4.1 | 2.0 |
| Shopping | 76.8 | 5.3 | 4.0 | 4.6 | 2.6 | 4.7 | 2.0 |
| Food Recipes | 71.5 | 8.6 | 7.3 | 3.3 | 2.0 | 5.4 | 2.0 |
| Comments on the News of the Day | 72.8 | 9.9 | 4.0 | 2.6 | 2.6 | 6.1 | 2.0 |
| Trends | 72.2 | 6.0 | 9.3 | 1.3 | 3.3 | 6.1 | 2.0 |
| Live Streaming | 84.1 | 3.3 | 3.3 | 1.3 | 1.3 | 4.7 | 2.0 |
| Memes | 29.8 | 13.2 | 18.5 | 11.9 | 13.9 | 11.4 | 1.3 |
| Dating | 78.1 | 6.0 | 7.9 | 1.3 | - | 4.7 | 2.0 |
| Sexual | 84.1 | 5.3 | 0.7 | - | 0.7 | 7.4 | 2.0 |
| Volunteering/Social Causes | 45.7 | 11.3 | 17.2 | 11.3 | 4.6 | 8.1 | 2.0 |
| Tourism Recommendations | 65.6 | 7.9 | 8.6 | 5.3 | 4.6 | 6 | 2 |

## How often do you post on social media?

| **Platform** | **Valid, n (%)** | **Less than Once a Week, n (%)** | **Approximately Once a Week, n (%)** | **Once Every 2-3 Days, n (%)** | **Once a Day, n (%)** | **Several Times a Day, n (%)** |
| --- | --- | --- | --- | --- | --- | --- |
| TikTok | 4 (2.6) | 140 (92.7) | 4 (2.6) | 1 (0.7) | 0 (0) | 2 (1.3) |
| Instagram | 1 (0.7) | 101 (66.9) | 24 (15.9) | 14 (9.3) | 6 (4.0) | 5 (3.3) |
| WhatsApp | 0 (0) | 56 (37.1) | 6 (4.0) | 5 (3.3) | 8 (5.3) | 76 (50.3) |
| YouTube | 3 (2.0) | 135 (89.4) | 3 (2.0) | 1 (0.7) | 2 (1.3) | 7 (4.6) |
| LinkedIn | 4 (2.6) | 145 (96.0) | 1 (0.7) | 0 (0) | 1 (0.7) | 0 (0) |
| Twitter | 4 (2.6) | 145 (96.0) | 1 (0.7) | 0 (0) | 0 (0) | 1 (0.7) |
| Facebook | 3 (2.0) | 139 (92.1) | 7 (4.6) | 0 (0) | 2 (1.3) | 0 (0) |
| Other | 3 (2.0) | 131 (86.8) | 9 (6.0) | 1 (0.7) | 5 (3.3) | 2 (1.3) |

*Note: n refers to the number of participants. Percentages may not add up to 100% due to rounding.*

Now, let's comment on this data:

The table provides a comparative overview of the posting frequency on different social media platforms. The highest frequency of "several times a day" is observed on WhatsApp, with over half of the participants (50.3%) reporting this level of usage. LinkedIn and Twitter show similar patterns, with the majority of participants (96.0%) posting less than once a week. The pattern for Facebook and YouTube is similar to LinkedIn and Twitter, but with slightly less frequency in the "less than once a week" category. Instagram displays a more evenly distributed posting frequency, although "less than once a week" is still the dominant category. Lastly, TikTok and other platforms show diverse usage patterns, but "less than once a week" remains the most common frequency. This data reveals that WhatsApp is the most frequently used platform for daily posting among the studied participants, whereas LinkedIn and Twitter are used least frequently.

## Do you think that social networks have changed you in the last 2 years:

| **Aspect** | **Completely FALSE (%)** | **2 (%)** | **3 (%)** | **4 (%)** | **5 (%)** | **6 (%)** | **Completely TRUE (%)** |
| --- | --- | --- | --- | --- | --- | --- | --- |
| Independence | 20.5 | 17.9 | 9.9 | 13.2 | 14.6 | 5.3 | 17.2 |
| Decision Making | 18.5 | 21.2 | 11.9 | 15.9 | 14.6 | 3.3 | 14.6 |
| Bonding with Friends | 23.8 | 16.6 | 12.6 | 13.9 | 10.6 | 5.3 | 17.2 |
| Tolerance Towards Sexual Minority Groups | 23.8 | 12.6 | 11.3 | 13.2 | 8.6 | 7.3 | 23.2 |
| Tolerance of Religious Minority Groups | 32.5 | 14.6 | 9.9 | 11.9 | 6.6 | 7.9 | 15.9 |
| Involvement in Community Problems | 18.5 | 15.9 | 14.6 | 19.9 | 10.6 | 7.9 | 11.9 |
| Creativity | 9.9 | 11.3 | 16.6 | 19.9 | 16.6 | 10.6 | 15.2 |
| Ability to Collaborate | 19.2 | 15.2 | 13.9 | 19.9 | 12.6 | 7.9 | 11.3 |
| Way of Eating | 25.2 | 15.9 | 9.3 | 14.6 | 15.9 | 8.6 | 10.6 |
| Socialization | 18.5 | 13.9 | 11.3 | 15.9 | 16.6 | 8.6 | 15.2 |
| Selection of Mentors | 29.1 | 16.6 | 13.2 | 13.9 | 13.9 | 2.6 | 10.6 |
| Self-Evaluation | 15.9 | 17.2 | 9.9 | 13.2 | 16.6 | 7.3 | 19.9 |
| Involvement in Online Communities | 32.5 | 14.6 | 15.9 | 13.9 | 7.9 | 4.6 | 10.6 |
| Ability to Concentrate | 24.5 | 11.3 | 9.3 | 12.6 | 12.6 | 7.9 | 21.9 |

## Descriptives on main variables:

| **Variables** | **N** | **Mean** | **Median** | **Mode** | **Std. Deviation** | **Minimum** | **Maximum** |
| --- | --- | --- | --- | --- | --- | --- | --- |
| SM Adiction | 151 | 15.245 | 15.00 | 14.00 | 5.618 | 6.00 | 30.00 |
| PHQ | 151 | 6.086 | 6.00 | 12.00 | 3.729 | 0.00 | 12.00 |
| SM Motives: Dating | 151 | 1.815 | 1.00 | 1.00 | 1.153 | 1.00 | 5.33 |
| SM Motives: New Friendships | 151 | 2.753 | 2.67 | 1.00 | 1.529 | 1.00 | 7.00 |
| SM Motives: Academic purposes | 151 | 4.449 | 4.67 | 7.00 | 1.759 | 1.00 | 7.00 |
| SM Motives: Social Conectedness | 151 | 4.263 | 4.33 | 7.00 | 1.802 | 1.00 | 7.00 |
| SM Motives: Following Others | 151 | 3.821 | 4.00 | 3.00 | 1.595 | 1.00 | 7.00 |
| SM Motives: Entertainment | 151 | 4.980 | 5.33 | 7.00 | 1.590 | 1.00 | 7.00 |
| SM Motives: Social Recognition | 151 | 2.041 | 1.67 | 1.00 | 1.137 | 1.00 | 6.00 |
| SM Motives: Self Expression | 151 | 2.863 | 2.67 | 1.00 | 1.562 | 1.00 | 7.00 |
| SM Motives: Seeking Information | 151 | 4.859 | 5.00 | 7.00 | 1.556 | 1.00 | 7.00 |
| Values: Conformity | 151 | 3.613 | 4.00 | 4.00 | 1.197 | 1.00 | 6.00 |
| Values: Tradition | 151 | 3.934 | 4.00 | 4.50 | 1.053 | 1.50 | 6.00 |
| Values: Benevolence | 151 | 5.232 | 5.50 | 6.00 | 0.690 | 2.50 | 6.00 |
| Values: Universalism | 151 | 5.190 | 5.33 | 5.67 | 0.679 | 2.67 | 6.00 |
| Values: Self_Direction | 151 | 4.867 | 5.00 | 5.00 | 0.745 | 2.50 | 6.00 |
| Values: Stimulation | 151 | 4.381 | 4.50 | 4.50 | 1.036 | 2.00 | 6.00 |
| Values: Hedonism | 151 | 4.599 | 4.50 | 5.00 | 0.917 | 1.00 | 6.00 |
| Values: Achievement | 151 | 4.530 | 4.50 | 4.00 | 1.020 | 1.50 | 6.00 |
| Values: Power | 151 | 3.580 | 3.50 | 4.00 | 1.041 | 1.00 | 6.00 |
| Values: Security | 151 | 4.825 | 5.00 | 5.00 | 0.863 | 2.00 | 6.00 |
| Values Cluster: Self Transcendence | 151 | 5.211 | 5.25 | 5.58 | 0.571 | 3.08 | 6.00 |
| Values Cluster: Self Enhancement | 151 | 4.055 | 4.00 | 4.00 | 0.875 | 1.75 | 6.00 |
| Values Cluster: Openess to Change | 151 | 4.616 | 4.67 | 5.00 | 0.658 | 2.83 | 6.00 |
| Values Cluster: Conservatorism | 151 | 4.124 | 4.17 | 4.33 | 0.763 | 2.00 | 5.83 |
|  |  |  |  |  |  |  |  |

## Sociodemographics:

| **Variable** | **Frequency (%)** | **Valid Percent (%)** | **Cumulative Percent (%)** |
| --- | --- | --- | --- |
| Year (1.00) | 104 (68.9%) | 68.9% | 100% |
| Mediu (2.00) | 129 (85.4%) | 85.4% | 100% |
| Language (en) | 140 (92.7%) | 92.7% | 100% |
| Module (Romanian module) | 137 (90.7%) | 90.7% | 92.1% |
| Gender (Female) | 104 (68.9%) | 68.9% | 68.9% |
