# Supplementary material for: Personal values clusters and their associations to social media behaviors and psychological well-being
Source: BMC Psychol. 2024 Oct 8;12:545. doi: 10.1186/s40359-024-02046-4 (PMC11462701; doi:10.1186/s40359-024-02046-4)
Supplement: Supplementary file 8 [file 40359_2024_2046_MOESM1_ESM.zip › Bergen scale.odt]

Here are six statements to consider. For each, answer: (1) very rarely, (2) rarely, (3) sometimes, (4) often, or (5) very often.

- You spend a lot of time thinking about social media or planning how to use it.

- You feel an urge to use social media more and more.

- You use social media in order to forget about personal problems.

- You have tried to cut down on the use of social media without success.

- You become restless or troubled if you are prohibited from using social media.

- You use social media so much that it has had a negative impact on your job/studies.
