# Supplementary material for: Personal values clusters and their associations to social media behaviors and psychological well-being
Source: BMC Psychol. 2024 Oct 8;12:545. doi: 10.1186/s40359-024-02046-4 (PMC11462701; doi:10.1186/s40359-024-02046-4)
Supplement: Supplementary file 8 [file 40359_2024_2046_MOESM1_ESM.zip › Scala_PVQ-21item.odt]

1. Thinking up new ideas and being creative is important to him/her. He/she likes

to do things in her own original way. SD

2. It is important to him/her to be rich. He/she wants to have a lot of money and

expensive things. PO

3. He/she thinks it is important that every person in the world be treated equally.

He/she believes everyone should have equal opportunities in life. UN

312

4. It's very important to him/her to show his/her abilities. He/she wants people to

admire what he/she does. AC

5. It is important to him/her to live in secure surroundings. He/she avoids

anything that might endanger his/her safety. SE

6. He/she likes surprises and is always looking for new things to do. He/she

thinks it is important to do lots of different things in life. ST

7. He/she believes that people should do what they're told. He/she thinks people

should follow rules at all times, even when no-one is watching. CO

8. It is important to him/her to listen to people who are different from him/her.

Even when he/she disagrees with them, he/she still wants to understand them.

UN

9. It is important to him/her to be humble and modest. He/she tries not to draw

attention to herself. TR

10. Having a good time is important to him/her. He/she likes to “spoil”

him/herself. HE

11. *It is important to him/her to make his/her own decisions about what he/she

does. He/she likes to be free and not depend on others. SD

12. It's very important to him/her to help the people around him/her. He/she

wants to care for their well-being. BE

13. *Being very successful is important to him/her. He/she hopes people will

recognize his/her achievements. AC

14. *It is important to him/her that the government insure his/her safety against all

threats. He/she wants the state to be strong so it can defend its citizens. SE

15. He/she looks for adventures and likes to take risks. He/she wants to have an

exciting life. ST

16. It is important to him/her always to behave properly. He/she wants to avoid

doing anything people would say is wrong. CO

17. *It is important to him/her to be in charge and tell others what to do. He/She

wants people to do what he/she says. PO

18. It is important to him/her to be loyal to his/her friends. He/she wants to

devote herself to people close to him/her. BE

19. He/she strongly believes that people should care for nature. Looking after the

environment is important to him/her. UN

20. *Tradition is important to him/her. He/she tries to follow the customs handed

down by his/her religion or his/her family. TR

21. He/she seeks every chance he/she can to have fun. It is important to him/her

to do things that give him/her pleasure. HE
