# Supplementary material for: Personal values clusters and their associations to social media behaviors and psychological well-being
Source: BMC Psychol. 2024 Oct 8;12:545. doi: 10.1186/s40359-024-02046-4 (PMC11462701; doi:10.1186/s40359-024-02046-4)
Supplement: Supplementary file 8 [file 40359_2024_2046_MOESM1_ESM.zip › Schita Chestionar SurveyMonkey.odt]

Survey  SurveyMonkey

1.Informarea si consimțământul după modelul de la studiul precedent cu de acord/nu sunt de acord si se incheie survey-ul

2.Ce varsta ai?

3.Ce gen ai?

Masculin/Feminin/Altul/Prefer sa nu spun

4.Esti la modul Romana sau Engleza? (aici putem pune toate scalele in romana pentru romani si le lasam in engleza pentru straini)

5.Ce an de studiu esti?

6.Mediu de proveniență - urban/rural 

7.Cat de numeros este anturajul tau? 

- Prieteni foarte buni

- Prieteni buni

8.Cât de des ai activități în comun cu 

- Familia

- Prietenii 

- Colegii 

9. Scala PVQ-21 (1 - Nu este deloc important 2 - Nu este foarte important 3 - Este relativ neimportant 4 - Este moderat de important 5 - Este important 6 - Este foarte important 7 - Este de o importanță extremă)

1. Thinking up new ideas and being creative is important to him/her. He/she likes

to do things in her own original way. SD

2. It is important to him/her to be rich. He/she wants to have a lot of money and

expensive things. PO

3. He/she thinks it is important that every person in the world be treated equally.

He/she believes everyone should have equal opportunities in life. UN

312

4. It's very important to him/her to show his/her abilities. He/she wants people to

admire what he/she does. AC

5. It is important to him/her to live in secure surroundings. He/she avoids

anything that might endanger his/her safety. SE

6. He/she likes surprises and is always looking for new things to do. He/she

thinks it is important to do lots of different things in life. ST

7. He/she believes that people should do what they're told. He/she thinks people

should follow rules at all times, even when no-one is watching. CO

8. It is important to him/her to listen to people who are different from him/her.

Even when he/she disagrees with them, he/she still wants to understand them.

UN

9. It is important to him/her to be humble and modest. He/she tries not to draw

attention to herself. TR

10. Having a good time is important to him/her. He/she likes to “spoil”

him/herself. HE

11. *It is important to him/her to make his/her own decisions about what he/she

does. He/she likes to be free and not depend on others. SD

12. It's very important to him/her to help the people around him/her. He/she

wants to care for their well-being. BE

13. *Being very successful is important to him/her. He/she hopes people will

recognize his/her achievements. AC

14. *It is important to him/her that the government insure his/her safety against all

threats. He/she wants the state to be strong so it can defend its citizens. SE

15. He/she looks for adventures and likes to take risks. He/she wants to have an

exciting life. ST

16. It is important to him/her always to behave properly. He/she wants to avoid

doing anything people would say is wrong. CO

17. *It is important to him/her to be in charge and tell others what to do. He/She

wants people to do what he/she says. PO

18. It is important to him/her to be loyal to his/her friends. He/she wants to

devote herself to people close to him/her. BE

19. He/she strongly believes that people should care for nature. Looking after the

environment is important to him/her. UN

20. *Tradition is important to him/her. He/she tries to follow the customs handed

down by his/her religion or his/her family. TR

21. He/she seeks every chance he/she can to have fun. It is important to him/her

to do things that give him/her pleasure. HE

Care este timpul mediu pe care îl petreceti pe urmatoarele retele sociale 

|  | 0-1h | 1,01-2h | 2,01-3h | 3,01-4h | 4,01-5h |
| --- | --- | --- | --- | --- | --- |
| Tik Tok |  |  |  |  |  |
| Instagram |  |  |  |  |  |
| WhatsApp |  |  |  |  |  |
| Youtube |  |  |  |  |  |
| LinkedIn |  |  |  |  |  |
| Twitter |  |  |  |  |  |
| Facebook |  |  |  |  |  |
| Altele ........ |  |  |  |  |  |

Care este timpul mediu pe care îl petreceti pe urmatoarele retele sociale 

|  | 0-1h | 1,01-2h | 2,01-3h | 3,01-4h | 4,01-5h |
| --- | --- | --- | --- | --- | --- |
| Tik Tok |  |  |  |  |  |
| Instagram |  |  |  |  |  |
| WhatsApp |  |  |  |  |  |
| Youtube |  |  |  |  |  |
| LinkedIn |  |  |  |  |  |
| Twitter |  |  |  |  |  |
| Facebook |  |  |  |  |  |
| Altele ........ |  |  |  |  |  |

Care este tipul de continut preferat pe care îl ACCESEZI frecvent pe retelele sociale ? (poti alege mai multe raspunsuri, unul sau niciunul, alegând )

(1 – foarte rar; 2 - rar; 3 – nici rar, nici des; 4 - des; 5 – foarte des; 6 – ns/nr)

| TIP CONȚINUT | 1 | 2 | 3 | 4 | 5 | 6 |
| --- | --- | --- | --- | --- | --- | --- |
| Noutăți despre prieteni aflați la distantă |  |  |  |  |  |  |
| Noutăți despre prietenii apropiați |  |  |  |  |  |  |
| Accesarea de profiluri noi |  |  |  |  |  |  |
| Comentarii politice |  |  |  |  |  |  |
| Informații educative |  |  |  |  |  |  |
| Recomandări despre sănătate |  |  |  |  |  |  |
| Informații religioase / spirituale |  |  |  |  |  |  |
| Tutoriale și ghiduri |  |  |  |  |  |  |
| Divertisment |  |  |  |  |  |  |
| Trenduri ale modei |  |  |  |  |  |  |
| Cumpărături |  |  |  |  |  |  |
| Retete de mancaruri |  |  |  |  |  |  |
| Știrile zilei |  |  |  |  |  |  |
| Comentarii despre stirile zilei |  |  |  |  |  |  |
| Recomandari |  |  |  |  |  |  |
| Live streaming |  |  |  |  |  |  |
| Influenceri |  |  |  |  |  |  |
| Memes |  |  |  |  |  |  |
| Dating |  |  |  |  |  |  |
| Sexual |  |  |  |  |  |  |
| Voluntariat / cauze sociale |  |  |  |  |  |  |
| Recomandări turistice |  |  |  |  |  |  |
|  |  |  |  |  |  |  |

Care este tipul de continut preferat pe care îl POSTEZI frecvent pe retelele sociale ? (poti alege mai multe raspunsuri, unul sau niciunul)

(1 – foarte rar; 2 - rar; 3 – nici rar, nici des; 4 - des; 5 – foarte des; 6 – ns/nr)

| TIP CONȚINUT | 1 | 2 | 3 | 4 | 5 | 6 |
| --- | --- | --- | --- | --- | --- | --- |
| Noutăti despre propria persoană |  |  |  |  |  |  |
| Comentarii politice |  |  |  |  |  |  |
| Informații educative |  |  |  |  |  |  |
| Recomandări despre sănătate |  |  |  |  |  |  |
| Informații religioase / spirituale |  |  |  |  |  |  |
| Tutoriale și ghiduri |  |  |  |  |  |  |
| Divertisment |  |  |  |  |  |  |
| Trenduri ale modei |  |  |  |  |  |  |
| Cumpărături |  |  |  |  |  |  |
| Retete de mancaruri |  |  |  |  |  |  |
| Comentarii despre stirile zilei |  |  |  |  |  |  |
| Recomandari |  |  |  |  |  |  |
| Live streaming |  |  |  |  |  |  |
| Memes |  |  |  |  |  |  |
| Dating |  |  |  |  |  |  |
| Sexual |  |  |  |  |  |  |
| Voluntariat / cauze sociale |  |  |  |  |  |  |
| Recomandări turistice |  |  |  |  |  |  |
|  |  |  |  |  |  |  |

Cât de frecvent postezi pe retelele sociale? 

|  | De mai multe ori pe zi | O data pe zi | O data la 2-3 zile | La cca. O saptamana | Mai rar de o data pe saptamana |
| --- | --- | --- | --- | --- | --- |
| Tik Tok |  |  |  |  |  |
| Instagram |  |  |  |  |  |
| WhatsApp |  |  |  |  |  |
| Youtube |  |  |  |  |  |
| LinkedIn |  |  |  |  |  |
| Twitter |  |  |  |  |  |
| Facebook |  |  |  |  |  |
| Altele ........ |  |  |  |  |  |

7.Scala pentru Motivatie SMU-SNS

The following statements are about the motives for using SNS. Read them and answer indicating to which extent they apply to you. 

| 1 | 2 | 3 | 4 | 5 | 6 | 7 |
| --- | --- | --- | --- | --- | --- | --- |
| Completely untrue |  |  |  |  |  | Completely true |

| 1. | To hook up | 1 | 2 | 3 | 4 | 5 | 6 | 7 |
| --- | --- | --- | --- | --- | --- | --- | --- | --- |
| 2. | To look for a date | 1 | 2 | 3 | 4 | 5 | 6 | 7 |
| 3. | To seek a romantic partner | 1 | 2 | 3 | 4 | 5 | 6 | 7 |
| 4. | To make new friends | 1 | 2 | 3 | 4 | 5 | 6 | 7 |
| 5. | To extend my circle of friends | 1 | 2 | 3 | 4 | 5 | 6 | 7 |
| 6. | To meet new people | 1 | 2 | 3 | 4 | 5 | 6 | 7 |
| 7. | To ask for information about what to study for the exams | 1 | 2 | 3 | 4 | 5 | 6 | 7 |
| 8. | To ask or share class notes | 1 | 2 | 3 | 4 | 5 | 6 | 7 |
| 9. | To check or share group assignments | 1 | 2 | 3 | 4 | 5 | 6 | 7 |
| 10. | To not feel disengaged from the world | 1 | 2 | 3 | 4 | 5 | 6 | 7 |
| 11. | To feel connected with people | 1 | 2 | 3 | 4 | 5 | 6 | 7 |
| 12. | To feel socially integrated | 1 | 2 | 3 | 4 | 5 | 6 | 7 |
| 13. | To keep up-to-date with what my contacts are doing in their day-to-day life | 1 | 2 | 3 | 4 | 5 | 6 | 7 |
| 14. | To know the details of my friends’ lives | 1 | 2 | 3 | 4 | 5 | 6 | 7 |
| 15. | To snoop on people that I am interested in | 1 | 2 | 3 | 4 | 5 | 6 | 7 |
| 16. | To fill my free time | 1 | 2 | 3 | 4 | 5 | 6 | 7 |
| 17. | To kill time when I am bored | 1 | 2 | 3 | 4 | 5 | 6 | 7 |
| 18. | To entertain myself | 1 | 2 | 3 | 4 | 5 | 6 | 7 |
| 19. | To stand out from others | 1 | 2 | 3 | 4 | 5 | 6 | 7 |
| 20. | For other people to comment my posts | 1 | 2 | 3 | 4 | 5 | 6 | 7 |
| 21. | To check that other like my posts | 1 | 2 | 3 | 4 | 5 | 6 | 7 |
| 22. | To express my feelings and thoughts | 1 | 2 | 3 | 4 | 5 | 6 | 7 |
| 23. | To give my opinion on a topic | 1 | 2 | 3 | 4 | 5 | 6 | 7 |
| 24. | To discuss some subject (with other people) | 1 | 2 | 3 | 4 | 5 | 6 | 7 |
| 25. | To keep up about what happens in the world | 1 | 2 | 3 | 4 | 5 | 6 | 7 |
| 26. | To be informed about the news | 1 | 2 | 3 | 4 | 5 | 6 | 7 |
| 27. | To find information about the topics that I like and am interested in | 1 | 2 | 3 | 4 | 5 | 6 | 7 |

Intrebari care sa evalueze continutul cel mai frecvent primit de ei?

Crezi ca retelele sociale ti-au modificat in ultimii 2 ani :

|  |  |  |  |  |  |  |
| --- | --- | --- | --- | --- | --- | --- |
| Independența |  |  |  |  |  |  |
| Modul de luare a deciziilor |  |  |  |  |  |  |
| Modul de a lega prietenii |  |  |  |  |  |  |
| Toleranța față de grupurile minoritare sexuale |  |  |  |  |  |  |
| Toleranța față de grupurile minoritare religioase |  |  |  |  |  |  |
| Gradul de iImplicare în rezolvarea problemelor comunității |  |  |  |  |  |  |
| Creativitatea |  |  |  |  |  |  |
| Capacitatea de a colabora |  |  |  |  |  |  |
| Modul de a mânca |  |  |  |  |  |  |
| Modul de socializare |  |  |  |  |  |  |
| Alegerea persoanelor mentor |  |  |  |  |  |  |
| Modul de a evalua propria persoană |  |  |  |  |  |  |
| Gradul de implicare în comunități online |  |  |  |  |  |  |
| Capacitatea de concentrare pe  termen lung |  |  |  |  |  |  |
| Percepția securității |  |  |  |  |  |  |
| Timpul petrecut în relații offline |  |  |  |  |  |  |
|  |  |  |  |  |  |  |

În ultimele două săptămâni: 

|  | Foarte rar | Rar | Nici rar, nici des | Des | Foarte des | Ns/ nr |
| --- | --- | --- | --- | --- | --- | --- |
| ați fost deranjat de sentimente de tristețe, dezinteres sau lipsa de plăcere în activități? |  |  |  |  |  |  |
| ați fost deranjat de sentimente de anxietate, cum ar fi teama, îngrijorarea sau neliniștea |  |  |  |  |  |  |
| aceste probleme v-au afectat capacitatea de a funcționa, de a lucra sau de a vă îndeplini responsabilitățile zilnice |  |  |  |  |  |  |
| ați avut probleme în relațiile cu prietenii, familia sau colegii din cauza acestor probleme? |  |  |  |  |  |  |

8. Scala Bergen pentru adictie 

Here are six statements to consider. For each, answer: (1) very rarely, (2) rarely, (3) sometimes, (4) often, or (5) very often.

- You spend a lot of time thinking about social media or planning how to use it.

- You feel an urge to use social media more and more.

- You use social media in order to forget about personal problems.

- You have tried to cut down on the use of social media without success.

- You become restless or troubled if you are prohibited from using social media.

- You use social media so much that it has had a negative impact on your job/studies.

De asemenea putem evalua foarte pe scurt cu 4 intrebari (Chestionarul PHQ-4 nivele de anxietate si depresie) pentru a include factorul Well-Being

Over the last two weeks, how often have you been bothered by the following problems? (Not at all Several days More than half the days Nearly every day)

 Feeling nervous, anxious or on edge 0 1 2 3 

Not being able to stop or control worrying 0 1 2 3 

Feeling down, depressed or hopeless 0 1 2 3 

Little interest or pleasure in doing things 0 1 2 3

Total score is determined by adding together the scores of each of the 4 items. Scores are rated as normal (0-2), mild (3-5), moderate (6-8), and severe (9-12). Total score ≥3 for first 2 questions suggests anxiety. Total score ≥3 for last 2 questions suggests depression.
